# The Efficacy of Flupentixol-Melitracen in the Adjuvant Therapy of Ulcerative Colitis in the Chinese Population: A Meta-Analysis

**DOI:** 10.1155/2019/3480732

**Published:** 2019-02-28

**Authors:** Xiaoqian Zhou, Lei Zhao, Ping Yang, Yaxi Chen, Xiong Z. Ruan

**Affiliations:** ^1^Department of Gastroenterology, The First People's Hospital of Guiyang, 550000 Guiyang, China; ^2^Centre for Lipid Research & Key Laboratory of Molecular Biology for Infectious Diseases (Ministry of Education), Institute for Viral Hepatitis, Department of Infectious Diseases, The Second Affiliated Hospital, Chongqing Medical University, 400016 Chongqing, China; ^3^The Collaborative Innovation Center for Diagnosis and Treatment of Infectious Diseases (CCID), Zhejiang University, 310058 Hangzhou, China; ^4^John Moorhead Research Laboratory, Centre for Nephrology, University College London Medical School, Royal Free Campus, University College London, London NW3 2PF, UK

## Abstract

**Objectives:**

Our aim of this study is to compare the efficacy of flupentixol-melitracen in the adjuvant therapy of ulcerative colitis patients in the Chinese population.

**Methods:**

Both the RevMan 5.2 and the Stata 12.0 software are used in this study for analysis, and a fixed-effect model (the Mantel-Haenszel method) or a random-effect model (the DerSimonian and Laird method) is used to merge or aggregate the risk ratio (RR) and its 95% confidence intervals (CI) of included studies.

**Results:**

Eleven trials involving 654 ulcerative colitis patients (treated group: 328; control group: 326) were analyzed in this study. Significant differences (RR = 1.29, 95% CI = 1.20 to 1.40, *P* < 0.001) between patients were observed between the two groups.

**Conclusions:**

Our results suggested that the efficacy of flupentixol-melitracen in the adjuvant therapy of ulcerative colitis is better than traditional drug treatments.

## 1. Introduction

Ulcerative colitis (UC) is a chronic nonspecific inflammatory disease involving the colon and rectum [1, 2], and its pathogenesis may result from changes in the colonic environment and the patients' psychological state [3–5]. Because the etiology and pathogenesis of the disease have not been fully elucidated, the recurrence, delayed healing course, and lack of specificity in treatment have brought mental and psychological pressure to patients. Mental and psychological pressure also affects the patient's immune system, affecting rehabilitation. Therefore, the psychological state of ulcerative colitis patients has been emphasised [6, 7]. If psychosocial factors do contribute to the development of the disease, then the treatment of anxiety and depression may be an effective way to treat UC. Some researchers showed that antianxiety and depression drugs can help alleviate symptoms of UC [8].

Flupentixol-melitracen, a clinically common anxiolytic drug, has been shown to have a better effect in adjuvant therapy for common digestive disorders such as irritable bowel syndrome and functional dyspepsia. Whether the effect of flupentixol-melitracen in the adjuvant therapy of ulcerative colitis patients in the Chinese population is superior to other traditional drug treatments alone is controversial [9–12], so we conducted a meta-analysis of the data from the current study and we explored and assessed the differences between the effects of flupentixol-melitracen in adjuvant therapy compared to traditional drug treatment, by means of extracted data.

## 2. Methods

### 2.1. Data Sources

We use PubMed, Wanfang Data (in Chinese), Springer, Chinese National Knowledge Infrastructure (CNKI, in Chinese), Elsevier ScienceDirect, and Google Scholar for searching up to December 2016. The keywords “flupentixol-melitracen,” “adjuvant therapy,” “ulcerative colitis,” “study,” and “trial” were used.

### 2.2. Inclusion and Exclusion Standards of Studies

1. *Inclusion Criteria*. The references included in this study must be published in full text. All the included trials are randomized controlled trials (RCT). Patients included in the study were in line with ulcerative colitis clinical diagnostic criteria (the revised standards of the National Symposium on Chronic Non-infectious Intestinal Diseases of Taiyuan). The trials included a treated group using flupentixol-melitracen combined with traditional drugs (such as mesalazine) and a control group only using traditional drug treatments for ulcerative colitis patients (shown in Table 1). The studies have a standard evaluation criteria
2. *Exclusion Criteria*. The references excluded in this study are duplicate publications and nonrandomized controlled trials. Subjects were confirmed cases of nonulcerative colitis. The trial did not set up a control group. Subjects had a clear history of mental illness.

### 2.3. The Efficacy Standard of Treatments

1. *Cure*. Clinical symptoms disappeared. Colonoscopy revealed normal intestinal mucosa
2. *Significantly Effective*. Clinical symptoms disappeared, and the recurrence of colonoscopy revealed a disappearance of inflammation. The biopsy showed the single diffuse mononuclear cells
3. *Effective*. Clinical symptoms disappeared. Colonoscopy revealed mild inflammation of the intestinal mucosa or pseudopolypoid
4. *Invalid*. There was no improvement in clinical symptoms and endoscopic and pathological findings after treatment

### 2.4. Evaluation of Quality and Meta-Analysis Methods

The evaluation of the quality of each study in this study mainly used the Jadad score. At the same time, meta-analysis was completed in a random or fixed-effect model. For each study, we used risk ratio (RR) and its 95% confidence interval (95% CI) to consolidate and summarize the statistical analysis results. We also used the Mantel-Haenszel method in the fixed-effect model [13] to obtain the combined estimates and used the DerSimonian and Laid methods in the stochastic effect model to calculate OR and its 95% CI of each risk factor [14]. In addition, we tested Cochran's *Q* statistic to further evaluate heterogeneity within the study and between the studies [15]. Moreover, we also used *I*^2^ = 100% × (*Q*-df)/*Q* to further quantify the substantial effect of heterogeneity within the study and between studies on the meta-analysis [16]. The heterogeneity of the study mainly is reflected by *Q* statistics (*P* < 0.10) or *I*^2^ statistics (*I*^2^ > 50%), and then the random-effect model is used for meta-analysis. Otherwise, we used the fixed-effect model. We performed all the statistical analysis using Review Manager 5.2 and the Stata package v.12.0.

### 2.5. Evaluation of Publication Bias

We measured the asymmetry of the funnel by the natural logarithmic scale of the effect size, and we used Egger linear regression [17] to evaluate the publication bias.

## 3. Results

### 3.1. The Screening Process and General Characteristics of the Study

**Figure 1:**
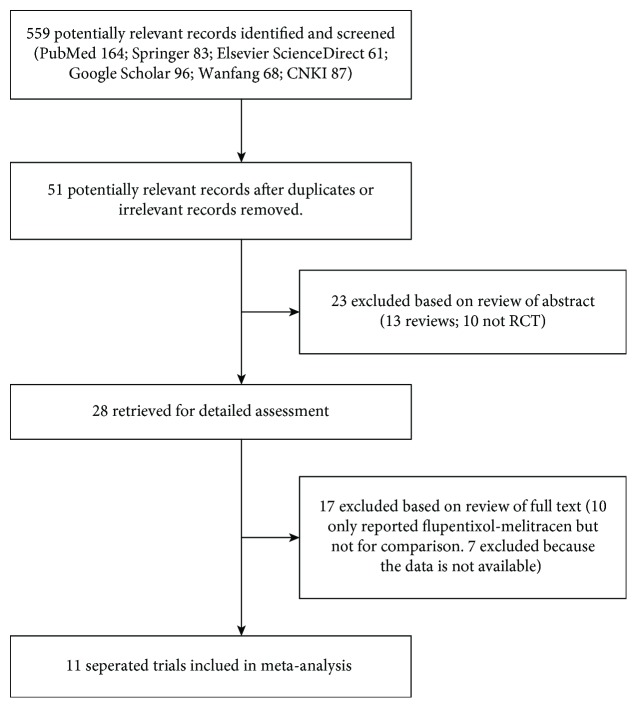
Flow diagram for selection of studies. 559 potentially relevant records identified and screened (PubMed: 164; Springer: 83; Elsevier ScienceDirect: 61; Google Scholar: 96; Wanfang Data: 68; CNKI:87); 51 potentially relevant records after duplicates or irrelevant records removed; 23 excluded based on the abstract (13 reviews; 10 not RCT); 28 retrieved for detailed assessment; 17 excluded based on review of the full text (10 only reported flupentixol-melitracen, but not for comparison; 7 due to nonavailable data); 11 separate studies included in meta-analysis.

559 references were related to this study (PubMed: 164; Springer: 83; Elsevier ScienceDirect: 61; Google Scholar: 96; Wanfang Data: 68; CNKI: 87). After the selection process by deleting irrelevant/duplicate records as shown in Figure 1, Fifty-one records were primarily involved in this study. Twenty-three of these records were removed by screening the abstracts (10 were not RCT; 13 were review articles). Finally, 28 trials were given a comprehensive review, and 11 trials were analyzed after deleting 17 unsuitable trials (10 only reported flupentixol-melitracen data, and 7 did not provide available data).

As is shown in Table 1, there are 11 trials [9–12, 18–24] in this study. The published years of recruited researches were between 2006 and 2015. A total of 654 ulcerative colitis patients (treated group: 328; control group: 326) were included in this study. The sample sizes of this study were between 40 and 80. The eleven trials were randomized controlled trials (RCT). Two had a Jadad score of 3 (Table 2), and nine had a Jadad score of 2.

### 3.2. Overall Efficacy of Flupentixol-Melitracen in the Adjuvant Therapy of Ulcerative Colitis

**Figure 2:**
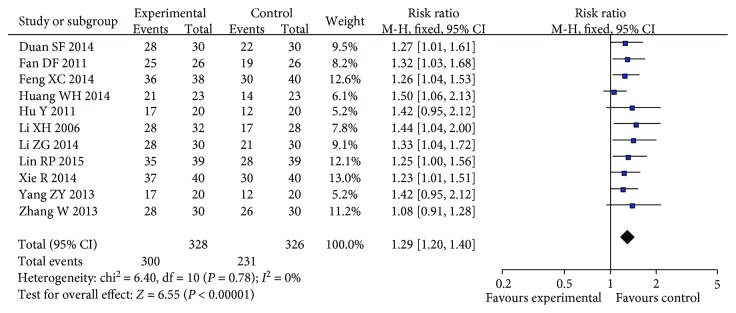
Forest plot of flupentixol-melitracen in the adjuvant therapy of ulcerative colitis with the experimental group vs. the control group.

The overall effects of flupentixol-melitracen in the adjuvant therapy of ulcerative colitis patients with this study are revealed in Figure 2. Eleven trials involving 654 ulcerative colitis patients with flupentixol-melitracen in the adjuvant therapy were analyzed in this study. No heterogeneities between studies (*Q*^2^ = 6.40, *I*^2^ = 0%, *P* > 0.1) were observed, so we used the fixed-effect model to compare efficacy between the treated group and the control group. Significant differences (RR = 1.29, 95% CI = 1.20 to 1.40, *P* < 0.001) in patients were observed between the two groups, suggesting that the efficacy of flupentixol-melitracen in the adjuvant therapy of ulcerative colitis in the treated group may be better than that in the control group.

### 3.3. Publication Bias Analysis Assessment

**Figure 3:**
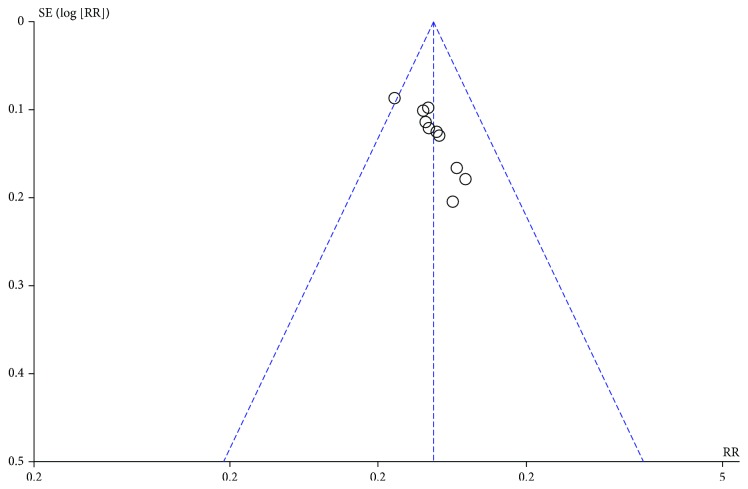
Funnel plot of flupentixol-melitracen in the adjuvant therapy of ulcerative colitis with the experimental group vs. the control group.

The almost symmetrical funnel plot (Figure 3) and Egger's linear regression test (*t* = −0.83, *P* > 0.05) of the flupentixol-melitracen efficacy in the adjuvant therapy of ulcerative colitis patients showed that there was no publication bias existing in our study.

## 4. Discussion

Some studies [11, 12, 20, 22, 23] assessed the effects of flupentixol-melitracen in the adjuvant therapy versus other traditional drug treatments for ulcerative colitis patients. But confusing results were found, as the sample sizes of the studies were not large enough and the statistical efficiency of the studies was relatively low. Thus, we combined the eleven trials which included the 654 ulcerative colitis patients (treated group: 328; control group: 326). Significant differences (RR = 1.29, 95% CI = 1.20 to 1.40, *P* < 0.001) in patients were observed between the control group and the treated group.

Ulcerative colitis (UC) is a chronic nonspecific intestinal inflammation. It mainly affects the mucosa and submucosa of the colon. It spreads from the distal colon to the proximal part of the colon and gradually affects the whole colon [25]. The clinical manifestations usually include abdominal pain, diarrhea, mucus pus, blood, and other symptoms. Symptoms may be persistent or recurrent and may be associated with the skin, mucous membranes, joints, and other parenteral performance. The current treatment of ulcerative colitis often uses 5-aminosalicylic acid drugs, hormones, immunosuppressive agents, and other drugs. Patients are symptomatic for a long period and the symptoms are recurrent. It has a great negative psychological impact on the patient and is often accompanied by depression, anxiety, and other symptoms, which will affect the effect of disease treatment.

Ulcerative colitis is a chronic inflammatory disease and has no special treatment [26]. The current application of anti-inflammatory drugs is mostly aminosalicylic acid, including mesalazine, 5-aminosalicylic acid, and sulfasalazine. The disease is a chronic gastrointestinal disease, which will lead to excessive diarrhea in patients. It can also lead to loss of nutritional ingredients in patients, which will endanger their lives. Therefore, it is essential to take effective treatment combined with the necessary antianxiety drug therapies to improve the patient's quality of life.

Research has shown that psychological factors alter brain-gut axis function, stimulating the autonomic nervous system; promote the release of various neurotransmitters; increase the activity of immune cells; and alter the interaction between bacteria and mucosa, which work together to damage the intestinal mucosa and lead to the occurrence of ulcerative colitis [27]. Flupentixol-melitracen is a compound preparation containing flupentixol and melitracen. Flupentixol acts on the D2 receptor, promotes the synthesis and release of dopamine, and increases the content of dopamine in synaptic space, which play an antianxiety and antidepression role. Melitracen is a biphasic antidepressant, which can inhibit the uptake of norepinephrine and serotonin through the presynaptic membrane inducing the increase of monoamine transmitters in synaptic space. The mixture of the two ingredients will combat depression and anxiety.

Although the included systematic studies were randomized controlled trials, the Jadad scores of the selected trials were 2 to 3, probably indicating a lower literature quality. Further studies with large-sample, multicenter, and prospective randomized trials are required to confirm the results of the current study.

## 5. Conclusions

Our results suggested that the efficacy of flupentixol-melitracen in the adjuvant therapy of ulcerative colitis in the experimental group is better than that in traditional drug treatments. The results of this study show that flupentixol-melitracen in the adjuvant therapy is a safe and effective treatment, although a multicenter and large-sample randomized controlled study is still needed to further confirm the results of this study.

## Figures and Tables

**Table 1:** Characteristics of studies included in the meta-analysis.

**S**tudy	Year of publication	Sample size	Sample size		Medicine		Study design	Age, years (mean ± SD or min-max)	Male
			Treated group	Control group	Treated group	Control group			
Li et al. 2006 [23]	2006	60	32	28	Mesalazine 1 g po qid; flupentixol-melitracen 10.5 mg po bid	Mesalazine 1 g po qid	RCT	26-64	39
Hu 2011 [22]	2011	46	23	23	Mesalazine 1 g po qid; flupentixol-melitracen 10.5 mg po bid	Mesalazine 1 g po qid	RCT	21-65	30
Fan 2011 [19]	2011	52	26	26	Mesalazine 1 g po qid; flupentixol-melitracen 10.5 mg po bid	Mesalazine 1 g po qid	RCT	NA	NA
Yang 2013 [24]	2013	40	20	20	Mesalazine 1 g po qid; flupentixol-melitracen 10.5 mg po bid	Mesalazine 1 g po qid	RCT	21-60	23
Hu 2013 [21]	2013	60	30	30	Mesalazine 1 g po qid; flupentixol-melitracen 10.5 mg po bid	Mesalazine 1 g po qid	RCT	24-52	NA
Duan 2014 [18]	2014	60	30	30	Mesalazine 1 g po qid; flupentixol-melitracen 10.5 mg po bid	Mesalazine 1 g po qid	RCT	22-59	16
Huang 2014 [9]	2014	40	20	20	Mesalazine 1 g po qid; flupentixol-melitracen 10.5 mg po bid	Mesalazine 1 g po qid	RCT	21-60	23
Xie et al. 2014 [11]	2014	80	40	40	Mesalazine 1 g po tid; flupentixol-melitracen 10.5 mg po bid	Mesalazine 1 g po tid	RCT	36.5 ± 11.2	32
Li 2014 [10]	2014	60	30	30	Mesalazine 1 g po qid; flupentixol-melitracen 10.5 mg po bid	Mesalazine 1 g po qid	RCT	23-75	35
Feng 2014 [20]	2014	78	38	40	Mesalazine 1 g po qid; flupentixol-melitracen 10.5 mg po bid	Mesalazine 1 g po qid	RCT	22-64	48
Lin 2015 [12]	2015	78	39	39	Mesalazine 0.2-0.3 mg/kg enema bid; flupentixol-melitracen 10.5 mg po bid	Mesalazine 0.2-0.3 mg/kg enema bid	RCT	26-69	42

**Table 2:** Jadad scoring items of each eligible study for meta-analysis.

Study	Was the study randomized?	Was the randomization method described and appropriate?	Was the study described as double-blind?	Was the method of blinding described and appropriate?	Was there a description of withdrawals and dropouts?	Jadad scores
Li et al. 2006 [23]	Yes	NA	NA	NA	Yes	2
Hu 2011 [22]	Yes	NA	NA	NA	Yes	2
Fan 2011 [19]	Yes	NA	NA	NA	Yes	2
Yang 2013 [24]	Yes	NA	NA	NA	Yes	2
Hu 2013 [21]	Yes	NA	NA	NA	Yes	2
Duan 2014 [18]	Yes	NA	NA	NA	Yes	2
Huang 2014 [9]	Yes	NA	NA	NA	Yes	2
Xie et al. 2014 [11]	Yes	Yes	NA	NA	Yes	3
Li 2014 [10]	Yes	NA	NA	NA	Yes	2
Feng 2014 [20]	Yes	NA	NA	NA	Yes	2
Lin 2015 [12]	Yes	Yes	NA	NA	Yes	3
